# Dr. W. R. Scott

**Published:** 1855-01

**Authors:** 


It is with the most sincere regret we announce the death of Dr. W. R. Scott, of Raleigh, N. C. He died suddenly about five weeks ago, and having been promised a biographical notice of him, we hoped to have received it in time for the present number of the Journal, but as it has not yet come to hand, we are compelled to defer its publication until our next issue. Dr. S. had resided in Raleigh for upwards of twenty years, and, both as a dentist and gentleman of high toned integrity, he enjoyed an enviable reputation. The elevation of the respectability of his profession ever constituted one of the most fondly cherished wishes of his heart. With a view to the promotion of this object, he some two or three years previous to his death, as we are informed, made his will, leaving his entire property to his Alma Mater, the Baltimore College of Dental Surgery. While we appreciate the motive that prompted him to this act of noble generosity, we would have greatly preferred, had it been the will of Providence, that he should have been spared for many years to come, as an ornament to the profession to which he was so sincerely and devotedly attached. His death will long be lamented by a large circle of friends. Although modest and unassuming in his pretensions, he was a most faithful and skillful practitioner. We shall ever fondly cherish the memory of this excellent man.

